# Baicalein Relieves Ferroptosis-Mediated Phagocytosis Inhibition of Macrophages in Ovarian Endometriosis

**DOI:** 10.3390/cimb44120422

**Published:** 2022-12-07

**Authors:** Zhi-Hui Yi, Shu-Qing Li, Jun-Ya Ke, Yun Wang, Ming-Zhi Zhao, Jing Li, Ming-Qing Li, Zhi-Ling Zhu

**Affiliations:** 1 Department of Obstetrics and Gynecology, Shanghai Medical College of Fudan University, Shanghai 200011, China; 2 Institute of Obstetrics and Gynecology, Hospital of Obstetrics and Gynecology, Fudan University, Shanghai 200080, China; 3 Key Laboratory of Female Reproductive Endocrine Related Disease, Hospital of Obstetrics and Gynecology, Fudan University, Shanghai 200080, China; 4 Department of Integrated Traditional & Western Medicine, Hospital of Obstetrics and Gynecology, Fudan University, Shanghai 200011, China

**Keywords:** macrophage, phagocytosis, ferroptosis, baicalein, endometriosis

## Abstract

Iron overload and oxidative stress have been reported to contribute to ferroptosis in endometriotic lesions. However, the possible roles of iron overload on macrophages in endometriosis (EMs) remain unknown. Based on recent reports by single-cell sequencing data of endometriosis, here we found significant upregulations of ferroptosis-associated genes in the macrophage of the endometriotic lesion. Additionally, there was an elevated expression of *HMOX1*, *FTH1*, and *FTL* in macrophages of peritoneal fluid in EMs, as well as iron accumulation in the endometriotic lesions. Notably, cyst fluid significantly up-regulated levels of intracellular iron and ferroptosis in Phorbol-12-myristate-13-acetate (PMA)-stimulated THP-1 cells. Additionally, high iron-induced ferroptosis obviously reduced PMA-stimulated THP-1 cells’ phagocytosis and increased the expression of angiogenic cytokines, such as vascular endothelial growth factor A (VEGFA) and interleukin 8 (IL8). Baicalein, a potential anti-ferroptosis compound, increased GPX4 expression, significantly inhibited ferroptosis, and restored phagocytosis of THP-1 cells in vitro. Collectively, our study reveals that ferroptosis triggered by high iron in cyst fluid promotes the development of EMs by impairing macrophage phagocytosis and producing more angiogenic cytokines (e.g., IL8 and VEGFA). Baicalein displays the potential for the treatment of EMs, especially in patients with high ferroptosis and low phagocytosis of macrophages.

## 1. Introduction

Endometriosis (EMs) is classically defined as the presence of endometrial glands and stroma outside the uterus and is an inflammatory, estrogen-dependent disease associated with pelvic pain and infertility [1]. It affects 10–15% of women of reproductive age [2]. However, diagnosis is often delayed by years, misdiagnosis is common, and effective treatment takes a long time [3]. In addition, while these symptoms can be alleviated with treatment, including hormone drugs or surgical lesion removal [4], patients have shown side effects, such as contraception with progestin drug treatment, and a 30–50% recurrence of EMs two to five years after surgery [5].

Ferroptosis as an iron-dependent form of non-apoptotic cell death [6], and it is closely linked to endometriosis attributed to its high iron environment. A recent study reported that overloaded iron in endometriosis peritoneal fluid (PF) had harmful effects on the development of early embryos [7]. Our recent study has reported that follicular fluid (EMFF) with iron overload induces ferroptosis of granulosa cells and oocyte dysmaturity, and further contributes to EM-related infertility [8]. Meanwhile, ferroptosis of ectopic endometrial stromal cells (EcESCs) is involved in the regulation of angiogenesis [9]. However, the effects of ferroptosis on the endometriotic immune microenvironment have not been investigated.

Macrophages, as the main immune cells in the peritoneal cavity, play an important role in inflammatory exacerbation and development of EMs. In addition, the macrophage of EMs with reduced phagocytosis fails to clear ectopic endometrial cells [10]. Our previous study has shown that high concentrations of heme in endometriotic peritoneal fluid significantly inhibit the phagocytosis of macrophages [11]. However, the detailed mechanism of impaired macrophage phagocytosis is largely unknown in EMs. Macrophage heterogenic data of endometriotic lesions were reported by single-cell RNA sequencing.

Baicalein (5,6,7-trihydroxyflavone) is a natural flavonoid compound extracted from scutellaria baicalensis Georgi (a traditional medicinal herb) [12]. In addition to antiviral [13], anti-inflammatory [14] and anti-cancer [15] bioactivities, baicalein also has anti-ferroptosis activity [16]. It has been reported that baicalein inhibits ferroptosis in neurons after traumatic brain injury [17]. Baicalein improves survival in mice exposed to systemic irradiation by inhibiting ferroptosis [18]. Baicalein also inhibits erastin-induced ferrous iron production, glutathione depletion, and lipid peroxidation in pancreatic cancer cells [16]. Our previous studies had shown that baicalein induced EcESCs apoptosis through the NF-kB signaling pathway [19] and inhibited the invasion of EcESCs by invasion-related proteins, including MT1-MMP, MMP2, and MMP9 [20].

In this study, therefore, we investigated the effects of iron-induced ferroptosis on the function of macrophages in endometriotic milium and explored the potential therapeutic effect of baicalein against iron-induced ferroptosis of macrophages in EMs in vitro.

## 2. Materials and Methods

### 2.1. Patients and Samples Collection

This study was approved by the Human Research Ethics Committee of Obstetrics and Gynecology Hospital, Fudan University (number 2019-103), and all participants provided their informed written consent. Ectopic endometrium samples, endometriotic PF, and cyst fluid were obtained by laparoscopy for ovarian EMs from 8 patients (age range, 25–39 years old) at the Obstetrics and Gynecology Hospital of Fudan University. Normal endometrium samples were collected from 8 patients (age range, 23–35 years old) who underwent dilatation and curettage. All cases were histologically confirmed in accordance with established criteria. Controlled PF was collected from women undergoing laparoscopic surgery for uterine fibroids or teratoma (*n* = 8). All the Ectopic endometrial samples (*n* = 8) collected in our study were in the proliferative phase of the cell cycle, and only stage I-II were based on the American Society for Reproductive Medicine (ASRM) criteria. All the endometrial samples, including normal endometrium samples and ectopic endometrial samples, were immediately fixed in 4% paraformaldehyde and then embedded in paraffin for Prussian blue staining. All the PF was used for the extraction of macrophages. Chocolate-like cystic fluid of ovarian endometriosis is stored at –80 °C after aseptic extraction. Subject group characteristics were listed in Table 1.

### 2.2. Cells Culture

The monocyte cell line THP1 was purchased from the ATCC collection. THP1 was pre-treated with phorbol-12-myristate 13-acetate (100 ng/mL; PMA, Sigma, Waltham, MA, USA) for 48 h for differentiation to macrophages.

### 2.3. Extraction of Macrophages

PF was centrifuged at 300× *g* at 4 °C for 10 min. Cell particles were treated at 4 °C for 5 min with Red Blood Cell Lysis Buffer (Beyotime, Shanghai, China) and cleaned twice with PBS. Then, human anti-CD14 microbeads (Miltenyi Biotec, Bergisch Gladbach, Germany) were used to select high-purity peritoneal macrophages according to the manufacturer’s protocols.

### 2.4. Total RNA Extraction and RT-qPCR

Total RNA was extracted from cultured cells by EZ-press RNA Purification Kit (EZ Bioscience, Wuhan, China), according to the manufacturer’s protocol. Firstly, mRNA was transcribed to cDNA (11141ES10, Yeasen Biotech, Shanghai, China) according to the manufacturer’s protocol. Quantitative PCR (qPCR) was performed using the Hieff UNICON^®^ Universal Blue qPCR SYBR Green Master Mix (Yeasen Biotech, Shanghai, China). The qRT-PCR primers are listed in Table 2. The mRNA expression of the samples was normalized using the 2−ΔΔCT method compared with ***ACTB***.

### 2.5. Gene Oncology (GO) Analysis, Kyoto Encyclopedia of Genes and Genomes (KEGG) Pathway Analysis

Gene ontology (GO) analysis [21] and pathway analysis have been down as reported [22].

### 2.6. Prussian Blue Staining

All endometrial samples were immediately fixed in 4% paraformaldehyde and then embedded in paraffin. Processed tissues were sliced into 5-μm sections. Slides were stained using Prussian Blue Dye Solution (Servicebio, Wuhan, China) according to the manufacturer’s protocols.

### 2.7. Intracellular Ferrous Iron Staining

FerroOrange (Fe2+ indicator) (MKbio, Shanghai, China) was dissolved in dimethyl sulfoxide (DMSO) to form a 1 mM solution, which was further diluted (1:200) to 5 μM working concentration. Macrophages were seeded in 12-well plates with different treatments. The cells were then washed twice in PBS and incubated in 5 μM FerroOrange solution for 30 min at 37 °C avoiding light. Finally, the cells were rinsed in PBS twice and immediately observed.

### 2.8. Analysis of Lipid Peroxidation

Macrophages were implanted in 6-well plates and then treated with cyst fluid solution and FeSO_4_ solution for a specified time. The cells were washed twice with PBS and stained with 10 μm C11-BODIPY (581/591) (Invitrogen, Waltham, MA, USA) for 30 min at 37 °C under shade.

### 2.9. Malondialdehyde (MDA) Content Assay

Malondialdehyde (MDA) was measured by Malondialdehyde (MDA) Content Assay Kit (Boxbio, Beijing, China). Differently treated cells were centrifuged to collect into centrifuge tubes. According to the number of cells (10^5^), 0.3 mL of extract liquid) was added to process the sample which was broken by μLtrasonic with an ice bath (Rate of 20% or 200 W, μLtrasound for 3 s, interval of 10 s, repeat for 30 times). The centrifuge tubes were centrifugated at 8000× *g* at 4 °C for 10 min. Then, the supernatant was put on ice for testing. Measuring tubes were added with 600 μL MDA testing working fluid, 200 μL Sample, 0 μL Distilled water, and 200 μL Reagent III. Blank tubes were added with 600 μL MDA testing working fluid, 0 μL Sample, 200 μL Distilled water, and 200 μL Reagent III. The tubes were treated with a boiling water bath for 60 min and then treated with an ice bath cooling to room temperature. The tubes were centrifugated at 10,000× *g* at room temperature for 10 min, and the supernatant was taken to measure. The light absorption values were measured at 450 nm, 532 nm, and 600 nm, calculating ∆A450 = A450 determination—A450 blank, ∆A532 = A532 determination—A532 blank, and ∆A600 = A600 determination—A600 blank.

### 2.10. Phagocytosis Assay

To assess phagocytosis efficiency, 100 µL of pre-treatment macrophages (106 cells/mL in PBS) was added into a 96-well plate, then 10 µL of opsonized FITC+ particles (10^7^ particles/mL) (Polyscience, Eppelheim, Germany) was added to the same 96-well plate and cultured for 30 min at 37 °C under shade. At the end of the 30 min incubation, cells were transferred from the 96-well plates to 1.5 mL EP tubes with 1 mL of cold PBS to stop the phagocytosis, then cells were washed twice with cold PBS. Finally, cells were resuspended in 500 µL of cold PBS and analyzed quickly using Flow CytoMetry.

### 2.11. Cell Viability Measurements

Macrophages were implanted in a 96-well plate and cultured for 24 h at 37 °C. After macrophages had undergone different treatments at specific times, the supernatant was removed, and then the cells were washed with PBS three times. 100 μL 1640 medium containing 10 μL Cell Counting Kit-8 (CCK-8, Byotime, Beijing, China) was added into each well. The cells were incubated for 1 h at 37 °C until they turned orange and then measured at an absorbance of 450 nm. All drugs and corresponding treatment concentration: erastin (HY-15763), 10 μM; RSL3 (HY-100218A), 5 μM Ferrostatin-1 (HY-100579), 10 μM; deferoxamine (HY-B0988), 200 μM; necrostatin (HY-15760) 2 μM; ZVAD-FMK(HY-16658B), 5 μM were purchased from MedChemExpress (MCE). FeSO_4_ 7H2O (F7002), 200 μg/mL; baicalein (465119), 20 μM were purchased from Sigma.

### 2.12. Statistical Analysis

All experiments were repeated in at least triplicate. All data were analyzed using GraphPad Prism version 9. All parameters were performed using an unpaired *t*-test, or Mann–Whitney, or one-way ANOVA. Data that were normally distributed are presented as the mean ± S.E.M. Statistical significance is indicated by *p* < 0.05.

## 3. Results

### 3.1. Ferroptosis-Related Genes Are Enriched in Macrophages of Endometriosis Lesions

**Figure 1:**
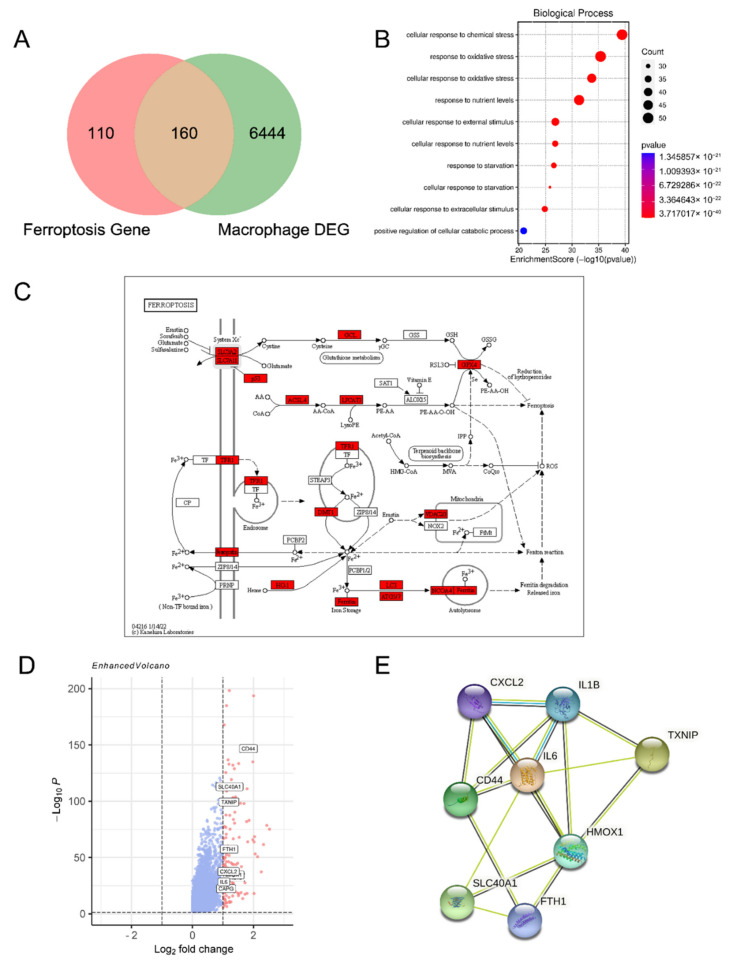
Identification of the ferroptosis-related genes in the macrophage of the endometriotic lesion. (**A**) Venn diagram to identify differentially expressed ferroptosis-related genes in macrophage of endometriotic lesions. (**B**) GO analysis showed the top 10 bubble plot of DE-FRGs. (**C**) DE-FRGs were labeled in the ferroptosis pathway. (**D**) The volcano plot represented total DEGs and showed ferroptosis-related genes with thresholds of log2FC ≥ 1. The horizontal line showed a *p*-value of 0.05 and the vertical lines represented the default 1.0-fold change. (**E**) The PPI network downloaded from the STRING database indicated the interactions among the DE-FRGs with thresholds of log2FC ≥ 1.

To investigate ferroptosis-related genes (FRGs) differentially expressed in macrophage in endometrioma, 270 FRGs were extracted from FerrDb (http://www.zhounan.org/ferrdb (accessed on 1 June 2021)), a database for drivers, suppressors, and markers associated with ferroptosis. Macrophage heterogenic data of endometriotic lesions were obtained from published data of single-cell RNA sequencing [23]. 6604 genes were significantly differentially expressed in macrophages of endometriosis compared with normal endothelium. After taking the intersection of differentially expressed genes (DEGs) and FRGs, a total of 160 FRGs expressed differentially were defined as DE-FRGs with thresholds of adjusted *p* < 0.05 (Figure 1A). Based on gene ontology (GO) analysis, the DE-FRGs were significantly enriched in the related processes, such as cellular response to chemical stress and response to oxidative stress (Figure 1B). The DE-FRGs were labeled in the ferroptosis-related pathway (https://www.kegg.jp/ (accessed on 1 June 2021)) (Figure 1C). As shown in Figure 1D, the volcano plot showed 9 DE-FRGs with thresholds of log2FC ≥ 1. Among these DE-FRGs, the PPI network was predicted by the STRING database (https://string-db.org/ (accessed on 1 June 2021)), showing that eight proteins (*CXCL2*, *IL1B*, *TXNIP*, *IL6*, *HOMX1*, *CD44*, *SLC40A1*, and *FTH1*) were closely related with ferroptosis (Figure 1E). As a key motivating factor in ferroptosis, high levels of iron metabolism-related genes like *HOMX1, SLC40A1,* and *FTH1* indicated there was iron accumulation in endometriotic macrophages.

### 3.2. Cyst Fluid Induces Iron Accumulation and Cell Death in Macrophage

**Figure 2:**
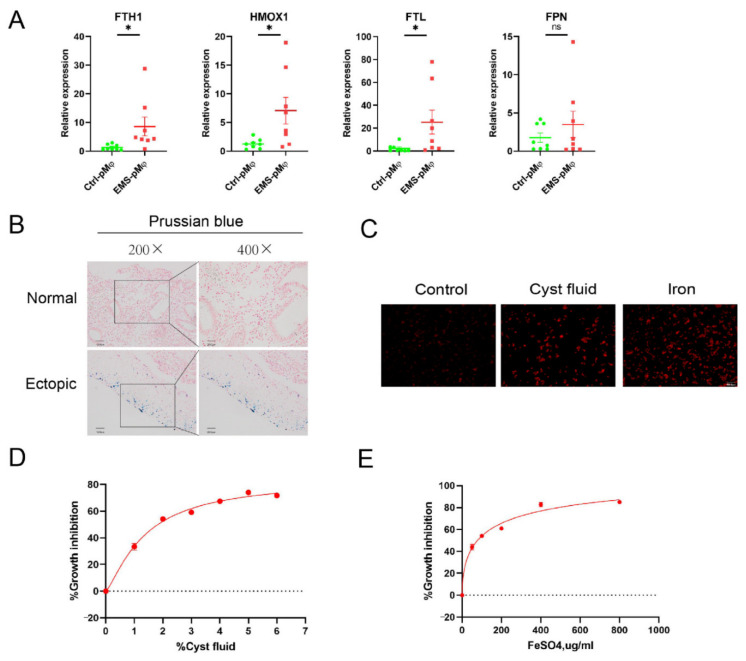
High iron level in cyst fluid induces cell death in macrophages. (**A**) Ctrl-pMϕ (*n* = 8) and EMs-pMϕ (*n* = 8) were extracted from the peritoneal fluid. The mRNA levels of *HMOX1*, *FTH1*, *FTL*, and *FPN* were measured with RT-qPCR. (**B**) Iron staining in normal endometrium (*n* = 8) and ectopic endometrium (*n* = 8). The scale bar = 100 μm for 200, 50 μm for × 400. (**C**) PMA-stimulated THP-1 cells were stained with FerroOrange after treatment of 2% cyst fluid and 200 μg/mL FeSO_4_ for 12 h. The scale bars = 100 μm. (**D**,**E**) PMA-stimulated THP-1 cells were treated with cyst fluid (0, 1, 2, 3, 4, 5, and 6 %) or FeSO_4_ solution (0, 50, 100, 200, 400, and 800 μg/mL) for 24 h. The cell viability was assayed using a CCK-8 kit. Iron: FeSO_4_; FTH1: ferritin heavy chain 1; HMOX1: heme oxygenase 1; FTL: ferritin light chain; FPN: solute carrier family 40 member 1; RT-qPCR: reverse transcription and quantitative real-time PCR. * *p* < 0.05.

Subsequently, we observed that the iron storage-related genes (e.g., *HMOX1*, *FTH1*, *FTL*) were highly expressed in the peritoneal macrophages of the EMs group, suggesting iron was accumulated in peritoneal macrophages in EMs (Figure 2A). To further validate local high iron in endometriosis, we used Prussian blue dye to stain the endometrial tissues. As shown, we observed iron accumulation in ectopic endometrial tissues, while there was no iron accumulation in normal endometrium (Figure 2B). To imitate the endometriotic microenvironment, we treated PMA-stimulated THP-1 cells with cyst fluid or FeSO_4_ solution. As shown, iron accumulation was clearly observed in the THP-1 cells after treatment with 2% cyst fluid and 200 μg/mL FeSO_4_ solution (Figure 2C). Of note, treatment with 2% cyst fluid and 200 μg/mL FeSO_4_ solution for 24 h clearly induced cell death of THP-1 cells significantly (Figure 2D,E). These data suggest that iron accumulation leads to cell death of macrophages in endometriosis.

### 3.3. Cyst Fluid Triggers Ferroptosis in Macrophages

**Figure 3:**
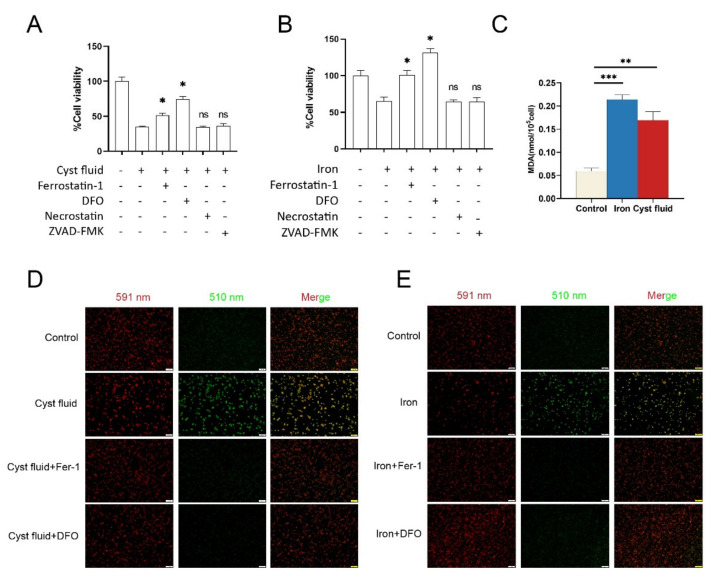
Cyst fluid-induced iron-dependent ferroptosis in macrophages. (**A**,**B**) PMA-stimulated THP-1 cells were treated with 2% cyst fluid or 200 μg/mL FeSO_4_ solution in the absence or presence of the indicated bioactive inhibitors (ferrostatin-1, 10 μM; DFO, 200 μM; necrostatin, 2  μM; ZVAD-FMK,5 μM) in 96-well plates for 24 h. Cell viability was measured with CCK-8 (* *p* <  0.05 vs. cyst fluid or FeSO_4_). (**C**) Intracellular MDA levels in THP-1 cells treated with 2% cyst fluid or 200 μg/mL FeSO_4_ solution. (**D**,**E**) Representative images of C11-BODIPY (581/591) staining in THP-1 cells with different treatments (cyst fluid,2%; FeSO_4_ solution, 200 μg/mL; ferrostatin-1, 10 μM; DFO, 200 μM). 591 nm shows the reduction state, and 510 nm shows the oxidation state. Scale bar = 100 µm. Fer-1: ferrostatin-1; DFO: deferoxamine. * *p* < 0.05, ** *p* < 0.01, *** *p* < 0.001.

To further investigate cell death induced by cyst fluid, we treated PMA-stimulated THP-1 cells with 2% cyst fluid and 200 μg/mL FeSO_4_ solution in the absence or presence of inhibitors that regulated cell death. The ferroptosis inhibitor ferrostatin-1, as well as the iron chelator deferoxamine (DFO), inhibited the death of THP-1 cells induced by cyst fluid and iron, while the apoptosis inhibitor ZVAD-FMK and necrostatin-1 failed to maintain the viability of THP-1 cells (Figure 3A,B). MDA, as one of the end products of lipid peroxidation, could also somewhat reflect the level of lipid ROS. The results of the MDA assay showed a significant increase in MDA levels in THP-1 cells after treatment with 2% cyst fluid and 200 μg/mL FeSO_4_ solution (Figure 3C). We also detected an obvious elevation of lipid ROS by C11-BODIPY fluorescent dye staining (Figure 3D,E), implying that iron in the endometriotic cyst triggers ferroptosis in macrophages.

### 3.4. Ferroptosis Reduces the Phagocytic Function of Macrophage

**Figure 4:**
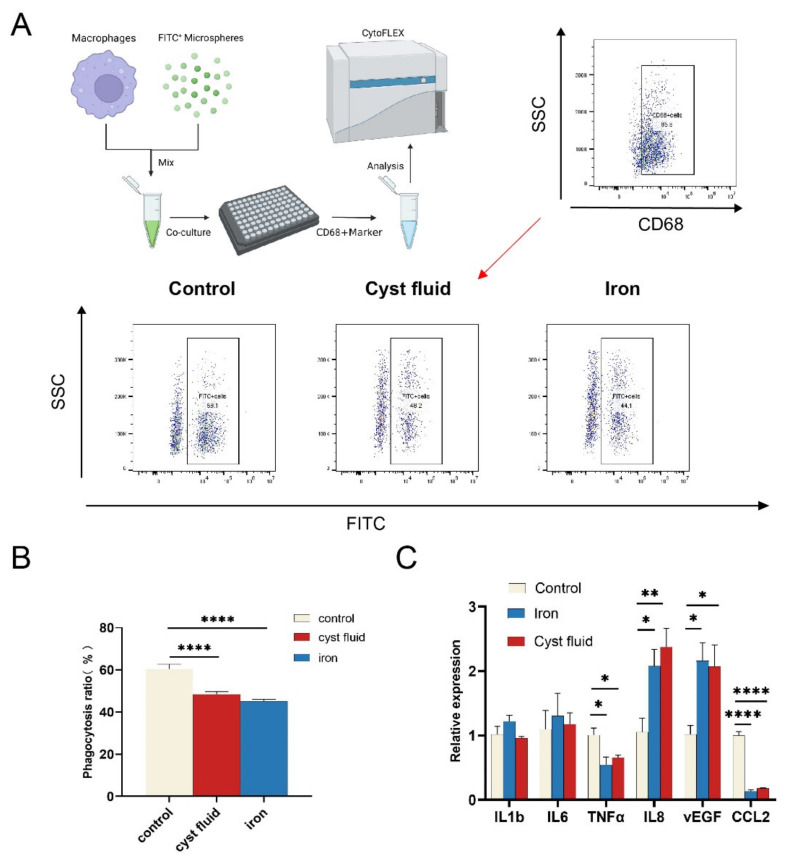
Effects of ferroptosis on macrophage function. (**A**) Representative image of phagocytosis ratio of macrophages treated with cyst fluid or iron verified by flow cytometric analysis. (**B**) Quantification of phagocytosis ratio of macrophages treated with cyst fluid or iron. (**** *p* < 0.0001). Phagocytosis ratio = FITC+CD68+cells/CD68+cells. (**C**) mRNA levels of cytokines expression in cyst fluid-treated (2%) and iron-treated (200 μg/mL) macrophages for 12 h determined by RT-qPCR. IL1b: interleukin 1 beta; IL6: interleukin 6; TNFα: tumor necrosis factor; IL8: C-X-C motif chemokine ligand 8; VEGFA: vascular endothelial growth factor A; CCL2: C-C motif chemokine ligand 2. * *p* < 0.05, ** *p* < 0.01, **** *p* < 0.0001.

To further explore the potential effects of high iron-induced ferroptosis on macrophage phagocytosis, PMA-stimulated THP-1 cells were treated with 2% cyst fluid and 200 μg/mL FeSO_4_ for 12 h, and then co-cultured with FITC+ particles at a certain concentration. As shown, pretreatment with cyst fluid or high iron markedly impaired the phagocytic ability of THP-1 cells (Figure 4A,B). More interestingly, we found that cyst fluid and high iron consistently upregulated the expression of *IL8* and *VEGFA* and downregulated the expression of *TNFα* and *CCL2* inTHP-1 cells (Figure 4C). However, there was no obvious change in *IL1b* and *IL6*. These findings indicate that ferroptosis should be involved in decreased phagocytosis and elevated angiogenesis of macrophages in the endometriotic milieu.

### 3.5. Baicalein Protects Macrophages against Ferroptosis and Rescues Phagocytosis

**Figure 5:**
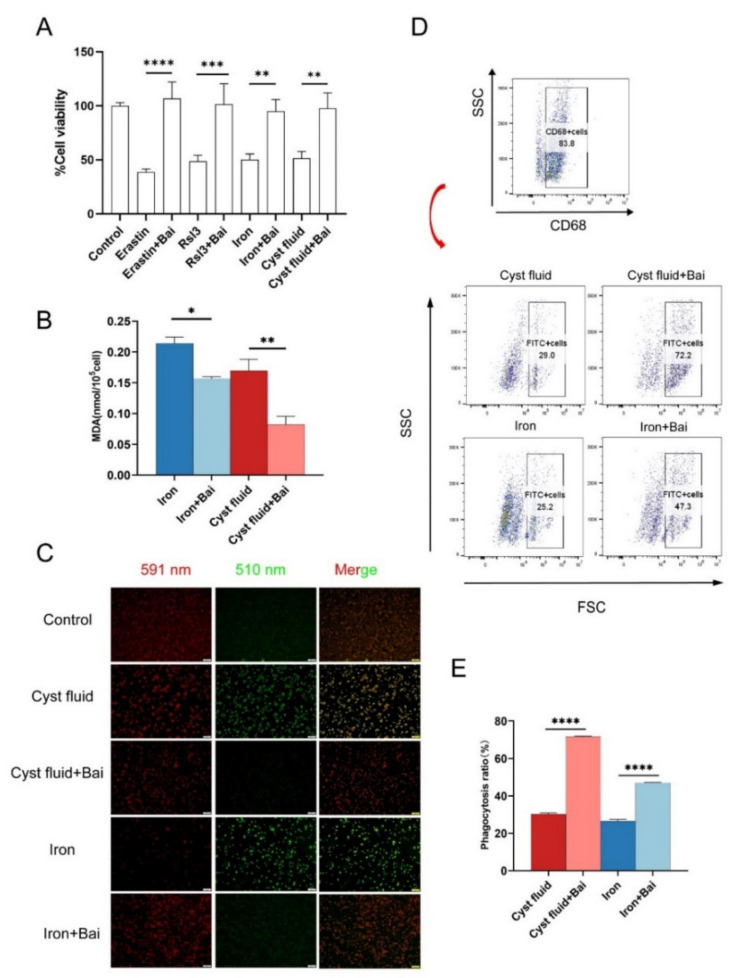
Baicalein inhibits macrophages’ ferroptosis and protects the phagocytic function. (**A**) PMA-stimulated THP-1 cells with different treatments as shown in the picture for 24 h (erastin, 10  μM; RSL3, 5 μM; cyst fluid, 2%; FeSO_4_ solution, 200 μg/mL; baicalein, 20 μM) in 96-well plates for 24  h. Cell viability was measured with CCK-8. (**B**) Intracellular MDA levels in THP-1 cells after incubation with 2% cyst fluid and 200 μg/mL FeSO_4_ in the absence or presence of baicalein for 12  h. (**C**) Representative images of C11-BODIPY (581/591) staining in THP-1 cells with different treatments (cyst fluid, 2%; FeSO_4_ solution, 200 μg/mL; baicalein, 20 μM). 591 nm shows the reduction state, and 510 nm shows the oxidation state. Scale bar = 100 µm. (**D**,**E**) Representative image of phagocytosis ratio of macrophages treated with cyst fluid and iron in the absence or presence of baicalein verified by flow cytometric analysis. Phagocytosis ratio = FITC+CD68+cells/CD68+cells. Bai: baicalein. * *p* < 0.05, ** *p* < 0.01, *** *p* < 0.001, **** *p* < 0.0001.

Baicalein is a natural compound with an anti-ferroptosis activity. Therefore, we further investigated the effect of baicalein on ferroptosis and the phagocytic function of macrophages. PMA-stimulated THP-1 cells were treated with cyst fluid, FeSO_4_, the ferroptosis inducers erastin and RSL3, and/or baicalein. The results showed that baicalein obviously inhibited cell death of THP-1 cells induced by cyst fluid, FeSO_4_, or ferroptosis inducers (Figure 5A). Baicalein also markedly inhibited MDA and lipid peroxide production induced by cyst fluid and iron (Figure 5B,C). These data suggest that baicalein restores macrophage phagocytosis in vitro (Figure 5D,E).

### 3.6. Baicalein Increases GPX4 Expression and Inhibits Ferroptosis in Macrophage

**Figure 6:**
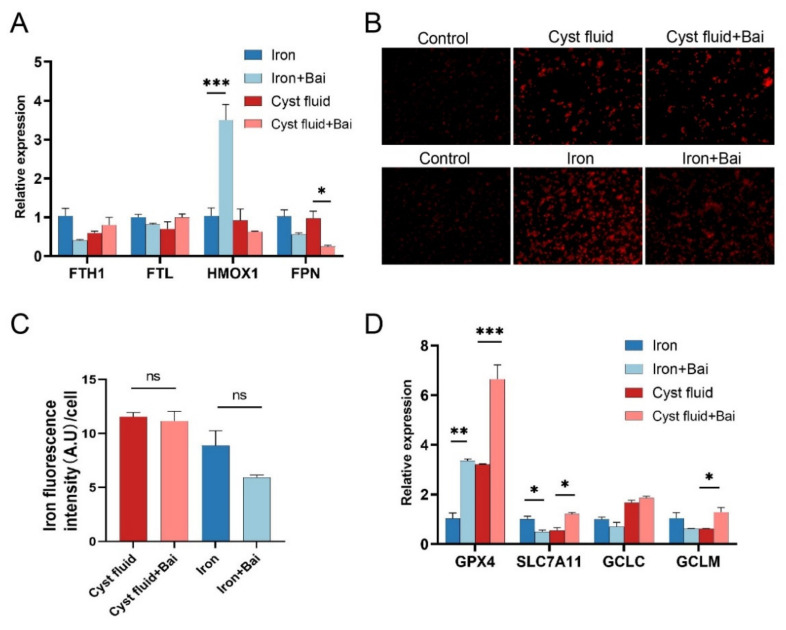
Baicalein promotes GPX4 expression but exerts no impact on iron transfer. (**A**) mRNA levels of iron metabolism-related genes expression in cyst fluid-treated (2%) and iron-treated THP-1 cells in the absence or presence of 20 μM baicalein for 12 h by RT-qPCR. (**B**,**C**) FerroOrange specific fluorescence in THP-1 cells after treatment of 2% cyst fluid and 200 μg/mL FeSO_4_ in the absence or presence of 20 μM baicalein for 12 h. The scale bars  =  100 μm. (**D**) mRNA levels of ferroptosis suppressor gene metabolism expression in cyst fluid-treated and iron-treated THP-1 cells for 12 h determined by RT-qPCR.GPX4: glutathione peroxidase 4; SLC7A11: solute carrier family 7 member 11; GCLC: glutamate-cysteine ligase catalytic subunit; GCLM: glutamate-cysteine ligase modifier subunit; A.U: arbitrary unit. * *p* < 0.05, ** *p* < 0.01, *** *p* < 0.001.

To investigate the possible mechanism by which baicalein inhibits ferroptosis, we analyzed the regulatory roles of baicalein on the transportation and metabolism of iron ions in macrophages. However, we did not find consistent alterations in iron metabolism-related molecules in THP-1 cells pretreated with cyst fluid and iron (Figure 6A). Similarly, we observed that baicalein did not affect cellular iron levels using iron probes (Figure 6B,C). Then, we detected the expression of system xc (-)-glutathione-GPX4 axis-related genes in THP-1 cells. It was found that baicalein up-regulated the mRNA expression of GPX4 in THP-1 cells pretreated with cyst fluid and iron (Figure 6D). These data indicate that baicalein increases GPX4 expression and elicits a protective effect against ferroptosis. And this effect is not dependent on iron ion transportation and metabolism.

## 4. Discussion

Macrophages in the abdomen fail to clear ectopic endometrium tissue, which is associated with reduced phagocytosis of macrophages in EMs [10]. It has been reported that co-culture with ectopic endometrial stromal cells reduces the phagocytosis of macrophages [24]. Chuang et al. have reported that prostaglandin E(2) inhibits the expression of CD36 in peritoneal macrophages, resulting in impaired phagocytic ability [25]. Estrogen-induced CD200 also inhibits macrophage phagocytosis, contributing to the immune escape of ectopic lesions [26]. High concentrations of heme significantly suppress the phagocytosis of macrophages [11]. However, the role of a high iron environment in macrophage phagocytosis is largely unclear. Here, we observed that high iron levels in cyst fluid have a significant negative impact on the phagocytosis of macrophages by inducing ferroptosis.

Angiogenesis is a prerequisite for the establishment and growth of endometriosis in vivo. It is physiologically dependent on macrophage activation, as depletion of macrophages jeopardizes VEGFA generation and angiogenesis in EM mice models [27,28]. A recent study revealed that VEGFA and IL-8 secretion were stimulated by ferroptosis of endometrial stromal cells and contributed to the angiogenesis of endometriotic lesions [9]. A similar result in our study has shown that cyst fluid and iron-induced ferroptosis can up-regulate *IL8* and *VEGF*A expression in THP-1 cells. Although ferroptosis has been found to occur in EMs, its role in the development of EMs has not been well explained. In our study, we found that cyst fluid and iron-induced ferroptosis in macrophages could trigger angiogenesis. Further mechanisms still need to be explored. PI3K/Akt and NF-kB pathways promote cell survival, the MAPK pathway promotes cell proliferation, and the ERK pathway promotes cell proliferation, survival, differentiation, migration, and angiogenesis. Through these signaling pathways, each member of the VEGF family provides different actions [29].

The number of peritoneal macrophages increased significantly in EMs, presenting with complex phenotypes. Numerous studies have shown a significant increase in macrophage populations and pro-inflammatory cytokines in PF and ectopic endometrium [30]. The therapeutic target of different immune subsets, such as tumor-associated macrophages, may be relevant to making progress in cancer immunotherapy, as well [31]. However, the phenotype of macrophages in endometriosis has not been fully characterized. Macrophages in endometriotic lesions have long been described as wound healing and “M2-like”, but few studies have considered the complexity of macrophage phenotype, where pro-inflammatory and wound healing-like markers often co-exist in response to complex signals from the local tissue microenvironment [32]. The association between macrophage phenotype complexity and high iron levels in EMs has not been systematically investigated. Iron can shape M1-like macrophage polarization through mitogen-activated protein kinase (MAPK), NF-κB, and other cellular signaling pathways, including ATF4, ROS, and NLRP3 inflammasome [33,34,35,36,37]. Conversely, iron supplementation also inhibits M1-like polarization by inhibiting STAT1 activation [38]. Iron supplementation down-regulates actin-regulatory protein glia maturation factor-γ (GMFG), which is negatively correlated with mitochondrial ROS (mtROS) accumulation [39]. Therefore, iron supplementation or overload affects macrophage polarization depending on various cellular pathways. The possible reasons for these different effects of iron on macrophage polarization are mainly related to the iron status of cellular iron (e.g., marginal or high iron supplementation) and the heterogeneity of the tissue microenvironment [31]. In addition, ferroptosis has a significant effect on macrophage polarization. In a recent study, autophagy-dependent ferroptosis via the release of oncogenic KRAS protein drove tumor-associated macrophage polarization [40]. Another study discovered that inducible nitric oxide synthase (iNOS)/NO-enrichment of activated M1, rather than alternatively activated M2 macrophages, modulates susceptibility to ferroptosis [41]. This result suggests that the high expression of M2 in ectopic lesions may be due to the role of high iron environment selectivity. In this study, we had not systematically investigated the effects of iron on macrophage polarization, but we found there were higher expressions of IL8 and VEGF, lower expressions of TNFα and CCL2, and no change in IL1b and IL6 in the macrophages treated with cyst fluid and iron. This observation suggests that high iron differentiates macrophages towards the phenotype with the activity of tissue repair and angiogenesis. Therefore, the complexity of iron and ferroptosis in macrophage polarization in endometriosis deserves further study.

Our previous studies have shown that baicalein induces EcESCs apoptosis and inhibits invasion [19,20]. We intended to explore more mechanisms of baicalein as a potential anti-ferroptosis drug in Ems. Baicalein is well known as a selective 12/15-LOX inhibitor. 12/15-LOX is a key enzyme that produces lipid peroxide [42], while GPX4 is responsible for reducing lipid peroxide [43]. The models of antagonistic interaction between 12/15-LOX and GPX4 have been described previously [44,45]. Many studies had proven baicalein’s effects on GPX4 and alox12/15 [46,47,48]. In this study, we also observed the anti-ferroptosis of baicalein on the macrophage, and GPX4 should be involved in this process. However, few studies focused on whether the effect of baicalein on GPX4 was via its inhibition of 12/15-LOX to date. The exact mechanisms of the anti-ferroptosis function of baicalein still have to be precisely elucidated.

**Figure 7:**
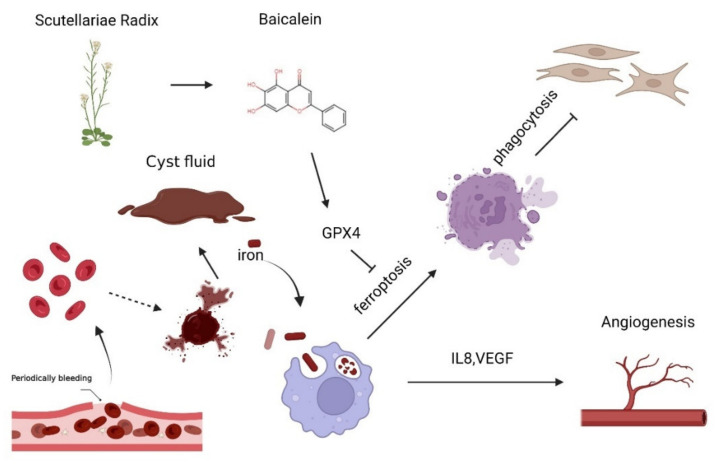
Baicalein restores ferroptosis-induced phagocytosis inhibition of macrophage in endometriosis. A high iron environment leads to ferroptosis of macrophages in ovarian endometriosis. Ferroptosis reduced phagocytosis in macrophages, thus leading to loss of ability to clear the ectopic endometrium. Additionally, the up-regulation of angiogenic factors *VEGFA* and *IL8* in macrophages in ferroptosis may contribute to angiogenesis in EMs. As a natural flavonoid compound extracted from scutellariae radix, baicalein effectively restores the reduced phagocytosis in macrophages induced by ferroptosis partly by increased GPX4 expression.

In conclusion, as depicted in Figure 7, high iron in cyst fluid induces ferroptosis, on the one hand, as well as directly reducing macrophage phagocytosis; on the other hand, it promotes the expression of angiogenic factors VEGFA and IL8 in macrophages, possibly contributing to the development of EMs together. Baicalein suppresses ferroptosis-mediated inhibition of macrophage phagocytosis as a potential anti-ferroptosis compound and is expected to contribute to the treatment of endometriosis.

## Figures and Tables

**Table 1:** Subject group characteristics—endometriosis (EMS), control PF (CP), and control endometrium (CE).

Variables Mean (Full Ranges)	EMS (*n* = 8)	CP (*n* = 8)	CE (*n* = 8)	*p* Value (EMS vs. CP)	*p* Value (EMS vs. CE)
Age, years	31 (25–39)	29.5 (21–44)	30.25 (23–35)	0.7053	0.7429
Gravidity, *n*	0.875 (0–2)	0.625 (0–2)	0.25 (0–1)	0.5797	0.0851
Parity, *n*	0.5 (0–1)	0.375 (0–1)	0.25 (0–1)	0.5773	0.4383
BMI, kg/m^2^	21.94 (17.99–25.64)	21.19 (18.14–27.85)	23.90 (19.95–30.84)	0.6059	0.2349
Phase (proliferative phase/secretory phase)	8/0	5/3	8/0	–	–

**Table 2:** The sequence of the primers for mRNAs.

Gene Name	Forward and Reverse Primer	Tm (℃)	Product Length (bp)
*ACTB*	F: 5′CTACCTCATGAAGATCCTCACC3′	60.8	250
	R: 5′AGTTGAAGGTAGTTTCGTGGAT3′	60.2	
*HMOX1*	F: 5′AAGACTGCGTTCCTGCTCAAC3′	62.9	247
	R: 5′AAAGCCCTACAGCAACTGTCG3′	62.6	
*FTL*	F: 5′CAGCCTGGTCAATTTGTACCT3′	60.0	114
	R: 5′GCCAATTCGCGGAAGAAGTG3′	62.0	
*FTH1*	F: 5′CCCCCATTTGTGTGACTTCAT3′	60.2	180
	R:5′GCCCGAGGCTTAGCTTTCATT3′	62.8	
*SLC40A1*	F: 5′CTACTTGGGGAGATCGGATGT3′	60.4	176
	R: 5′CTGGGCCACTTTAAGTCTAGC3′	60.1	
*GPX4*	F: 5′GAGGCAAGACCGAAGTAAACTAC3′	60.6	100
	R: 5′CCGAACTGGTTACACGGGAA3′	61.8	
*SLC7A11*	F: 5′TCTCCAAAGGAGGTTACCTGC3′	61.1	123
	R: 5′AGACTCCCCTCAGTAAAGTGAC3′	60.5	
*GCLC*	F: 5′GGAGGAAACCAAGCGCCAT3′	62.7	79
	R: 5′CTTGACGGCGTGGTAGATGT3′	61.9	
*GCLM*	F: 5′TGTCTTGGAATGCACTGTATCTC3′	60.0	239
	R: 5′CCCAGTAAGGCTGTAAATGCTC3′	60.7	
*IL1b*	F: 5′ATGATGGCTTATTACAGTGGCAA3′	60.0	132
	R: 5′GTCGGAGATTCGTAGCTGGA3′	60.8	
*IL6*	F: 5′ACTCACCTCTTCAGAACGAATTG3′	60.2	149
	R: 5′CCATCTTTGGAAGGTTCAGGTTG3′	61.3	
*TNFα*	F: 5′CCTCTCTCTAATCAGCCCTCTG3′	60.8	220
	R: 5′GAGGACCTGGGAGTAGATGAG3′	60.2	
*IL8*	F: 5′TTTTGCCAAGGAGTGCTAAAGA3′	60.1	194
	R: 5′AACCCTCTGCACCCAGTTTTC3′	62.5	
*CCL2*	F: 5′CAGCCAGATGCAATCAATGCC3′	62.3	190
	R: 5′TGGAATCCTGAACCCACTTCT3′	60.4	
*VEGFA*	F: 5′AGGGCAGAATCATCACGAAGT3′	61.2	75
	R: 5′AGGGTCTCGATTGGATGGCA3′	62.9	

Tm: temperature. bp: base pair.

## Data Availability

Not applicable.
